# Influence of mangrove forests on subjective and psychological wellbeing of coastal communities: Case studies in Malaysia and Indonesia

**DOI:** 10.3389/fpubh.2022.898276

**Published:** 2022-11-10

**Authors:** Guek-Nee Ke, I. Ketut Aria Pria Utama, Thomas Wagner, Andrew K. Sweetman, Aziz Arshad, Tapan Kumar Nath, Jing Yi Neoh, Lutfi Surya Muchamad, Djoko Santoso Abi Suroso

**Affiliations:** ^1^Department of Psychology, School of Social Sciences, Heriot-Watt University Malaysia, Putrajaya, Malaysia; ^2^Department of Naval Architecture, Institute Teknologi Sepuluh Nopember, Surabaya, Indonesia; ^3^The Lyell Centre, Heriot-Watt University, Edinburgh, United Kingdom; ^4^Department of Aquaculture, Faculty of Agriculture, Universiti Putra Malaysia, Serdang, Malaysia; ^5^School of Environment and Geographical Sciences, University of Nottingham Malaysia, Semenyih, Malaysia; ^6^Bandung Institute of Technology, Bandung, Indonesia

**Keywords:** mangrove ecosystems, socio-economic, psychological wellbeing, coastal communities, subjective wellbeing

## Abstract

Mangrove forests possess multiple functions for the environment and society through their valuable ecosystem services. Along with this, the mangrove forests have large and diverse social values, in combination contributing to the health and wellbeing of the surrounding communities. This study aims (i) to assess the benefits of mangrove forests and their impact on subjective and psychological wellbeing of coastal communities and (ii) to understand the challenges coastal communities face that limit sustainable wellbeing. We have used a mixed methodological approach, combining workshop, interview, and survey, to obtain qualitative and quantitative information from two coastal communities in Malaysia and Indonesia. For quantitative data, 67 participants from both coastal communities participated using a pre-tested structured questionnaire. To obtain opinions from key informants in Malaysia and Indonesia, we organized two stakeholders' workshops and community interviews. When merging these interviews and workshops, we identified the following three themes related to the perception of mangrove forest benefits: (1) the advantage of living in a natural countryside; (2) the natural resources supporting employment, income, and family security; and (3) the increase in subjective and psychological wellbeing. The mean score of wellbeing for Indonesian participants (28.6) was slightly higher than that for Malaysian participants (26.2) and was significant. Overall, the respondents felt happy because the combination of job security and leisure activities supports feeling content and satisfied. The analyses also suggest that the combination of exposure to coastal environments and stress reduction promotes good mental health; however, diagnostic health data are lacking. The lower score of mental wellbeing in Malaysia is attributed to respondents involved in risky fishing activities and local regions with excessive tourism. The findings from this study imply that coastal mangrove forest management plays an important role in the living conditions of coastal communities and their subjective and psychological wellbeing. Hence, restoration and sustainability of mangrove ecosystem are important.

## Introduction

Mangrove forests possess multiple functions for the environment and society through their valuable ecosystem services, including provisioning, regulating, habitat, and cultural services. These unique forests bordering tropical coastlines worldwide (1–3) have high significance in terms of economy and ecological functions, for example, through provision of storm and tsunami protection for communities who live in coastal areas (4–7). The wide range of ecosystem services provided by mangrove forests have large and diverse social values, in combination contributing to the health and wellbeing of the surrounding communities (3, 8–12). The social value of mangroves is closely associated with deeply held historical, communal, ethical, religious, and spiritual attributes, which are considered as sources of subjective wellbeing (13).

Subjective wellbeing is a multidimensional construct capturing basic human psychological needs, such as security, materials supporting a satisfactory life, health, and successful social relationships (14). It is known as an umbrella that includes individual emotional responses, domain satisfactions, and global judgments of life satisfaction (15, 16). More specifically, subjective wellbeing refers to peoples' opinion and feelings of their surrounding natural environment that impact satisfaction on life and happiness (17, 18), which is considered a key indicator defining quality of life (19). Along with subjective wellbeing, the intrinsic values (e.g., aesthetic, moral, and cultural values) of ecosystem services and the interactions between human and nature have a bearing upon psychological wellbeing of community people (13, 20, 21). It is evidence that direct and indirect contact with nature improves peoples' emotion, reduces stress, makes them feel more alive and cooperative, and thus improves psychological wellbeing of people (22, 23). Various studies have shown [e.g., (24, 25)] that forest-based activities such as forest walk and viewing scenic beauty have positive impacts on mental health, including stress, anxiety, depression, negative emotions, and quality of life.

There has been substantial research on the linkages between subjective wellbeing and nature in many countries, particularly in western nations, including Australia, East Asia, European countries, and North America (26, 27). More specifically, within the mangrove forest context, subjective wellbeing refers to a measure that assesses the relationship of individuals with the forest. Such studies are, however, very limited in Southeast Asia like Indonesia and Malaysia (28). Current understanding emphasizes that understanding subjective wellbeing related to ecosystems services is crucial for balancing a good life while supporting sustainable development (29), and hence, researchers have suggested to include the notion of subjective wellbeing in natural resources management (27). Maintaining and promoting a congenial relationship between forest ecosystem services and subjective wellbeing is an important aspect of regional sustainable development and hence is considered essential (30–32). A comprehension of subjective wellbeing may help to address forest management problems commonly faced by policymakers (28).

To the best of our knowledge, no previous studies on subjective wellbeing effects of forests or nature specifically explored the impact of mangrove forests on psychological wellbeing. As mentioned above, having these research gaps, this study aimed (i) to assess benefits of mangrove forests and their impact on subjective and psychological wellbeing of coastal communities and (ii) to understand the challenges toward sustainable wellbeing of coastal communities. Data for this study were collected from two coastal communities in Malaysia and Indonesia. We approach subjective wellbeing as an assessment based on personal judgments of general happiness or satisfaction (33) and psychological wellbeing indicating the positive functioning of individuals in relation to life satisfaction (34).

## A conceptual framework of subjective and psychological wellbeing and mangrove forests

**Figure 1:**
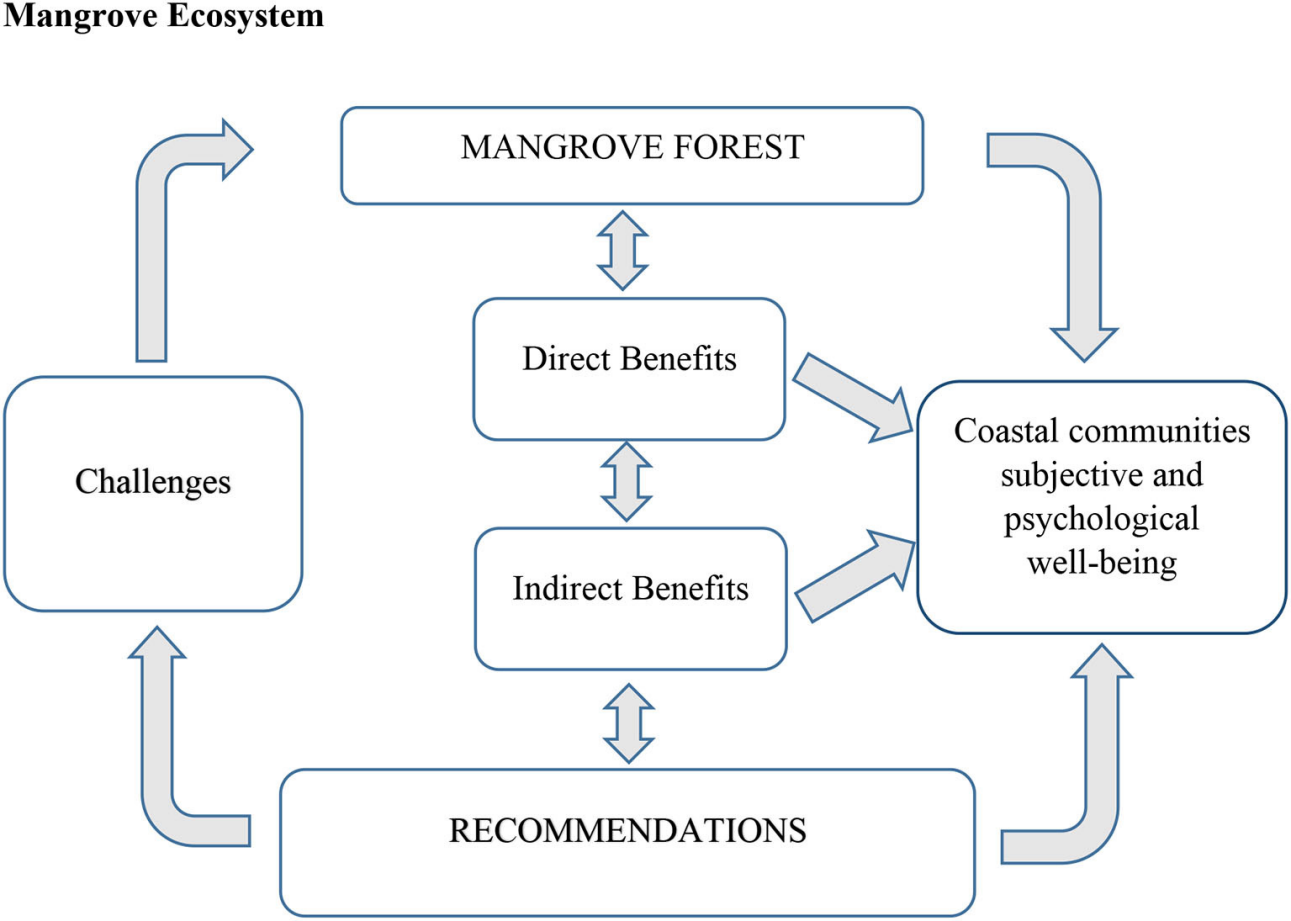
A conceptual framework showing possible relationships between mangrove forest systems and wellbeing of coastal communities.

Research to date has partly tested the benefits of mangrove ecosystem on subjective and psychological wellbeing of coastal communities. We assumed that mangroves through their direct (e.g., timber, firewood, fish, etc.) and indirect (e.g., protection from storms and flood, etc.) benefits influence both subjective and psychological wellbeing of coastal communities (Figure 1). In this study, SWB is comprised of two components, namely, feeling happy and satisfaction with life (15, 35). Satisfaction with life evaluates how the environment, income, livelihood, and work–life balance impact the subjective wellbeing of people (36–38). Different from that, psychological wellbeing of local people combines feeling confident, relaxed, cheerful, optimistic about future, and close to other people, all linked to their connectivity to mangrove forests (39–41). Mangrove ecosystems are subject to many challenges (e.g., degrade the ecosystem), which need action toward sustainability and improved management. In this study, we collect new information on the relationship between mangrove forests and local peoples' wellbeing and anticipate that they can support recommendations for sustainable forests management to the benefits of the coastal communities.

## Study areas

Two mangrove sites were selected based on the richness of the mangrove ecosystem in the two Southeast Asia countries of Malaysia and Indonesia.

***Matang Mangrove Forest Reserve (MMFR)*** is located in the north-west coast of the Peninsular of Malaysia (4°51′7.14″N−100°38′48.50″E). The Perak State Forestry Department manages MMFR covering about 40,466 ha and still considered as the best managed mangrove forest in the world (42). MMFR management includes a healthy charcoal industry (43, 44) with Matang being responsible for 70% of international charcoal export from Malaysia in year 2013 (45). The second important commodity is tourism and education, including bird watching, forest, and biodiversity research (22, 46). These activities have improved the economic condition of local communities in the MMFR area, supported by strict management (47). Data for this study were collected from deliberately selected two villages, namely Kampung Baru Kuala Sepetang and Kampung Menteri, in MMFR.

***Mangrove Wonorejo Surabaya (MWS)*** is a protected mangrove area in Surabaya, Indonesia (7°18′76″S−112°48′ 922″ E to 7°18′328″ S−112°50′691″E). MWS covers ~700 ha and is managed by the Department of Food Security and Agriculture of the City of Surabaya Government and declared as a conservation area by the City Major of Surabaya under Surabaya City Regional Regulation No. 3, 2007 (48, 49). The mangrove ecosystems in Wonorejo are managed to increase human welfare, in sectors such as education, conservation, and rehabilitation (50). Eco-tourism in MWS attracts both local and outside visitors, which directly or indirectly develop and upgrade the welfare of local communities living in and around mangrove forests (51–54), through broad opportunities for economic development (55, 56). Data for this study were collected from Pamurbaya in MWS.

## Methods

This study adopted qualitative and quantitative methodologies to collect data.

### Qualitative study

We initiated two qualitative approaches, namely, stakeholder workshop and key informant interviews.

#### Stakeholder workshops

Researchers organized 2 full-day stakeholder workshops at MMFR and MWS in February 2020. The aim of the workshops was to understand the challenges and opportunities of mangrove forests management in both study sites. Representatives (23 in MMFR and 21 in MWS) from 22 organizations joined in the workshops, including: the Department of Forestry, Department of Fisheries, Perak Fishermen's Association, Fisheries Research Institute Malaysia, Malaysian Nature Society of Perak Branch, Maritime Institute of Malaysia, Malaysian Wood Industries Association, Wetlands International Malaysia, Forest Research Institute Malaysia, Department of Survey and Mapping (Perak), Regional Planning and Development Agency (East Java and Surabaya), Fishery and Marine Department of East Java, communities leaders, NGOs, universities, local administrations, and others.

#### Key informant interviews

Key informant interviews are qualitative in-depth interviews with a wide range of people who have first-hand knowledge about the coastal communities. We conducted 67 interviews with the coastal communities (e.g., community leaders, forest rangers, business owners, fisherman, charcoal factory workers, and related stakeholders) at both sites.

### Quantitative study

Quantitative data on socio-demographic characteristics and subjective and psychological wellbeing were collected from the respondents. We used a purposeful structured questionnaire for collecting quantitative data on subjective wellbeing of two core components to explore the relationship between experiences and reactions toward the mangrove ecosystem of coastal communities and their subjective wellbeing. For psychological wellbeing, we followed the Warwick–Edinburgh Mental Wellbeing Scale (SWEMWBS), which is being widely used to understand psychological wellbeing of community people (39–41, 57).

Through the community leaders, we interviewed 67 respondents (40 in MMFR and 27 in MWS) who agreed to participate. All interviews were conducted through phone due to mobility restrictions during COVID-19 pandemic. As English is not the first language of the respondents, the questions were initially developed in English and then translated to Malay, the main language used by the local communities in Matang, Malaysia, and Surabaya, Indonesia.

### Ethical approval

Ethical approval was obtained from the research ethics committee of the School of Social Sciences Ethics Committee, Heriot-Watt University. In addition, we secured respondents' verbal consent for participation and audio-recording for this study. All respondents remain anonymous in this study.

## Data analysis

All qualitative data from the interview transcripts were transcribed and translated into English for data analysis. We used the inductive approach of thematic analysis introduced by Braun and Clarke (58), which describes patterns across qualitative data by identifying, analyzing, and reporting themes within data. The respondents' responses were coded, and codes that had similar emerging patterns were grouped together to form a theme or sub-theme. The data were examined for differences and similarities both within and across themes.

For quantitative data, SWEMWBS scores from respondents were calculated to give a mean score within and between the population sample from Malaysia and Indonesia, respectively. Mean scores for MMFR and MWS were compared according to the score categorization suggested by Warwick Medical School (59, 60). Descriptive attributes (frequency, percentage, and mean) were reported for socio-demographic and psychological wellbeing responses. We conducted the Chi-squared test to find out association between socio-demographic characteristics (age and education) and responses on benefits of mangrove forests.

## Results and discussion

### Socio-demographic characteristics of respondents

Table 1 shows the socio-demographic characteristics of the respondents, separated for Malaysia and Indonesia. In both study sites, male respondents were higher (57.5 and 66.7% in MMFR and MWS, respectively). A majority of respondents in both sites obtained high school education. In terms of occupation, fishing-related employment dominated in MMFR, whereas the majority of the respondents in MWS were involved in ecotourism. The monthly household income of the respondents in MWS ranged from USD115 to USD268, which was by far exceeded at MMFR USD258–USD2484.

**Table 1:** Socio-demographic characteristics of respondents.

**Variable**	**MMFR**		**MWS**	
	**Frequency**	**Percentage**	**Frequency**	**Percentage**
**Gender**				
Female	17	42.5	9	33.3
Male	23	57.5	18	66.7
**Age (years) range**				
18–30	2	5	10	37.1
31–45	20	50	8	29.6
>45	18	45	9	33.3
**Education**				
No formal education	3	7.5	–	–
Elementary school	11	27.5	4	14.8
Middle school	15	37.5	6	22.2
High school	10	25	14	51.9
College	1	2.5	3	11.1
**Profession**				
Community leader	–	–	1	3.7
Fisherman	14	32.6	3	10.8
Food and beverages	–	–	11	39.3
Eco-tourism	4	9.3	11	39.3
Business	2	4.7	1	3.6
Unemployed	4	9.3	–	–
Other (services, house keeper, housewife, odd jobs)	15	34.9	1	3.6
Factory worker	1	2.3	–	–
Supplier	1	2.3	–	–
Forest ranger	1	2.3	–	–
Public servant	1	2.3	–	–
**Monthly income range based on USD (Indonesia**)
>268 (Very high)			13	48.2
193–268 (High)			7	25.9
115–192 (Standard)			6	22.2
<115 (Low)			1	3.7
**Monthly income range based on USD (Malaysia)**
>2,484	1	2.5		
1,126–2,483	2	5		
<258–1,125	37	92.5		

#### Respondents' perception on benefits of mangrove forests

Respondents in both MMFR and MWS reported several direct and indirect benefits from mangrove forests (Table 2). In MMFR, most of the respondents stated that mangrove forests are the source (direct benefits) of fish and charcoal (19.8%) and other sea food products (18.5%). For respondents in MWS, mangrove forests are important for running ecotourism activities (65.1%), followed by fish (32.6%). Respondents in both sites also reported several indirect benefits, including protection from storm, flood, and strong waves, soil and riverbank erosion prevention, natural beauty, and carbon sequestration (Table 2).

**Table 2:** The frequency distribution of benefits from mangroves in MMFR and MWS multiple responses.

**Benefits**	**MMFR**		**MWS**		**Mean of MMFR and MWS**
**Direct benefits**	**Frequency**	**%**	**Frequency**	**%**	
Timber	11	13.6	–	–	8.9
Pole	3	3.7	–	–	2.4
Fish	16	19.8	14	32.6	24.2
Water	2	2.5	–	–	1.6
Wild food	1	1.2	–	–	0.8
Tourism	8	9.9	28	65.1	29.0
Firewood	5	6.2	–	–	4.0
Charcoal	16	19.8	1	2.3	13.7
Other seafood product	15	18.5	–	–	12.1
Other non-timber forest products	4	4.9	–	–	3.2
**Indirect benefits**					
Protection from storms	28	25.2	15	22.7	24.3
Protection of riverbank	12	10.8	–	–	6.8
Flood protection	19	17.1	8	12.1	15.3
Improve fertility of agricultural land	1	0.9	–	–	0.6
Biodiversity conservation	4	3.6	6	9.1	5.7
Carbon sequestration	16	14.4	8	12.1	13.6
Space for spiritual functions	1	0.9	–	–	0.6
Natural beauty	18	16.2	15	22.7	18.6
Protection from strong waves and tsunami	12	10.8	4	6.1	9
Protect the beach from soil erosion	-	-	10	15.2	5.7

The thematic analysis of qualitative data revealed the following three themes: (1) the advantage of living in a natural countryside; (2) the natural resources supporting employment, income and family security; and (3) the increase in subjective and psychological wellbeing. Below, we outline some features of these three themes:

Theme 1: The advantage of living in a natural countryside

Respondent-013:“*I feel that it is more relaxing here because places like fishing village are always more relaxed.”* Furthermore, respondent-015: “*Since we are living in the countryside, the pace of living here is slower, not like in the city where the pace of living is faster*.”

This may illustrate the more laid-back lifestyle in MMFR compared with urban areas that leads to carefree spirit among local communities. Meanwhile, respondent-006 attributed the relaxed and carefree spirit among local communities to the improving economic situation in MMFR: “*I think you can see changes, changes from the economy in one family*.”

Theme 2: The natural resources supporting employment, income, and family security

Natural resources such as wood for charcoal and fisheries have provided support for the livelihood of local communities. Charcoal production has created economic opportunities for small businesses and job opportunities in factory. Many respondents sell charcoal and other products made from charcoal, such as wood vinegar for a living. Mangrove forests in MMFR provide a breeding ground for fisheries which actively contribute to the abundant fisheries resources. For example, shrimp is one of the main fisheries catch in Kuala Sepetang.

Respondents commented that “*we supply it (shrimp) to the wet market*” and “*it is only here and the nearby areas*.”

Local communities can secure basic needs for their daily life due to the improving economic situation in Matang. Most of the respondents showed satisfaction of their current earnings that can be mainly attributed the satisfaction toward being able to support their livelihood.

For instances, respondents commented that “*we are fine with current income sources, it's sufficient for villagers like us*” and “*It is enough for our spending*.” With Kuala Sepetang being located in the countryside,

Respondent-015 mentioned that “i*t is sufficient to support a living in countryside but not enough for a living at the city*.”

Mangrove forests are the natural habitat for many species that are important to keep the food cycle in the ecosystem (61) and hence provide income sources to coastal communities round the year. The positive perception of the local community in MMFR and MWS on mangrove ecosystems may be associated with many benefits provided by the forests itself.

Themes 3: The increase in subjective and psychological wellbeing

Respondents in MWS also mentioned that they felt happy because there are job security and leisure activities which provide an environment which supports a comfortable lifestyle.

Respondent-026 commented that her husband enjoyed this place as her husband loved fishing. Another respondent-021 also revealed: “*Tourism activities provide jobs for many of the local people in this area. We feel so happy because these jobs can make our economic status get better*.”

Mangroves contribute to the tourism industry with various activities to offer, including nature education center, place for bird or fireflies watching and river cruises (62). Eco-tourism in mangrove areas provides large opportunities for jobs and small business to improve livelihoods of coastal communities (7, 51, 53, 54) as well as economic development of surrounding areas (52, 55, 56).

### Subjective and psychological wellbeing

We investigated two main variables, namely, satisfaction with life and work and feeling happy, to assess the subjective wellbeing of respondents. We asked four questions related to satisfaction with life and work. The results show that more than 70% of the respondents were satisfied with their life and work in coastal areas (Table 3). Iqbal (3) reported that mangroves provide several important sources of income to coastal communities in Bangladesh and so local people are happy and satisfied with living in mangrove areas. Moreover, Jones et al. (63) found that people living near a protected area have higher subjective wellbeing level.

**Table 3:** Respondents' opinion on SWB of the benefits of mangrove forests in MMFR and MWS.

**Variable**	**MMER**		**MWS**		**Both sites (%)**
	**Frequency (Yes answer)**	**%**	**Frequency (Yes answer)**	**%**	
**Satisfaction with life and work**					
Are benefits of MMFR/MWS important to your livelihood?	29	73	27	100	87
Are you currently satisfied with your income?	35	87	26	96	92
Are you currently satisfied with your occupation?	16	40	27	100	70
Do you feel that your livelihood would be affected if you do not obtain benefits from mangrove forests?	40	100	27	100	100
**Feeling happy**					
Are you happy living in coastal areas and benefiting from mangrove forests?	24	60	22	81	71

The results of psychological wellbeing of respondents due to the presence of mangrove forests showed that the mean scores of SWEMWBS for Indonesian respondents (mean = 28.6, SD = 3.14) are higher than those for Malaysian respondents (mean = 26.29, SD = 4.37). A non-parametric (Mann–Whitney) test was conducted to examine the differences between mean scores for Malaysian and Indonesian respondents to accommodate for the unequal number of participants in both study areas. The analysis shows that the differences between mean scores of SWEMWBS for Indonesia and Malaysia were significant (*U* = 748.00, *z* = 2.67, *p* < 0.05).

**Figure 2:**
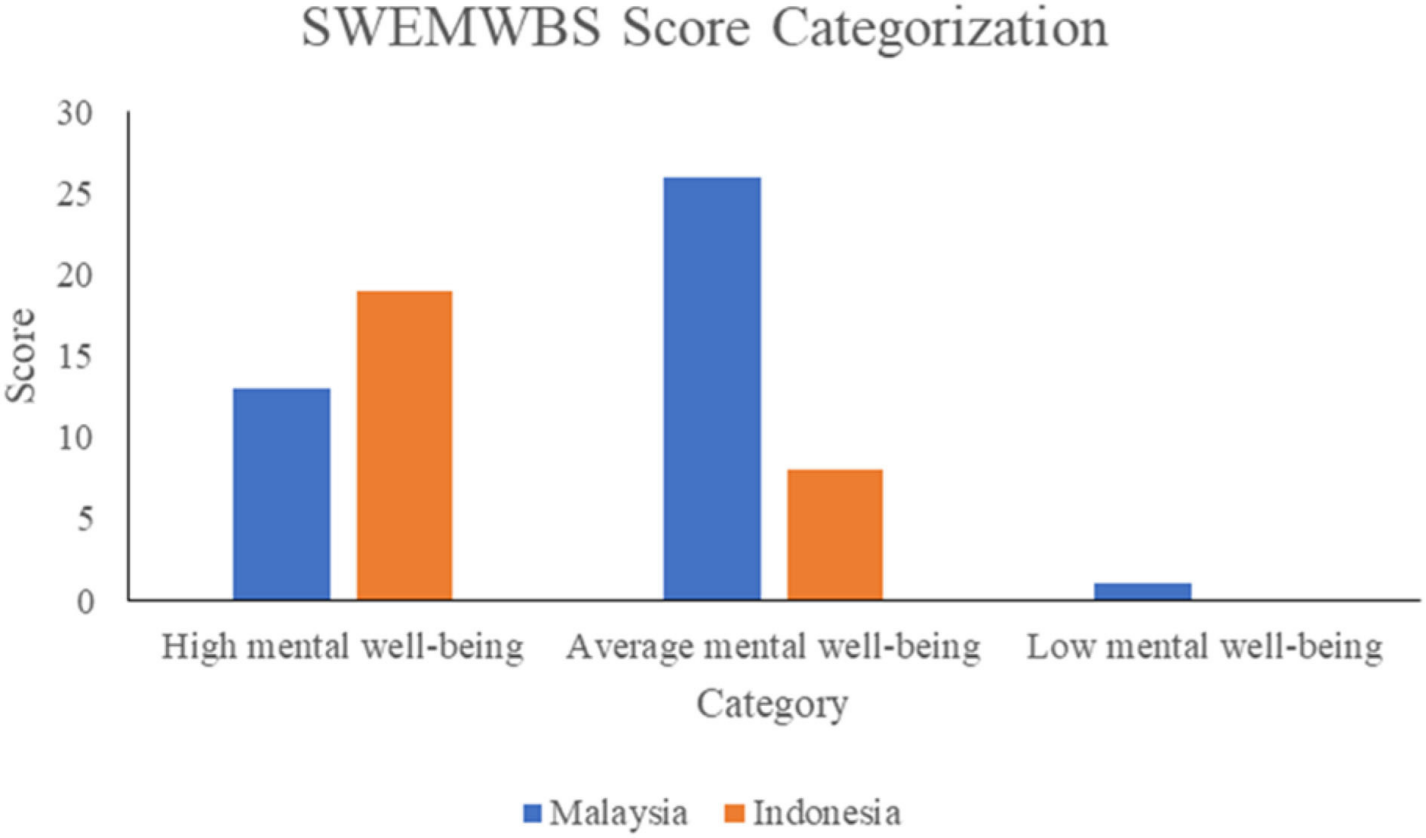
SWEMWBS score categorization of respondents in Malaysian and Indonesian sites.

Following SWEMWBS, the scores were categorized into high, average, and low mental wellbeing using the following cut-off points: high mental wellbeing (mean score 28–35), average mental wellbeing (20–27), and low mental wellbeing (7–19). Figure 2 illustrates the breakdown of SWEMWBS score categorization within each study site. Among Malaysian respondents, 33% reported having high mental wellbeing and 65% having average mental wellbeing. As for Indonesian respondents, 70% belonged to the high mental wellbeing category. However, we are cautious to draw a conclusion that Indonesian respondents were enjoying a high level of mental wellbeing because the number of interview respondents was small.

This outcome of mental wellbeing is associated with the neighborhood mangrove environment in which coastal communities of Matang and Wonorejo live. White et al. (64, 65) suggested that the association of exposure to coastal environments and stress reduction may promote good mental health. Grabowska-Chenczke et al. (23) commented that nature relatedness is a basic psychological need, which is strongly connected to affective and cognitive aspects of human wellbeing. Furthermore, environmental aspects of a coastal area may be described as an attractive, quiet, and peaceful settings, supporting high levels of mental wellbeing (66). In addition, a well-managed mangrove forest could bring psychological benefits in terms of identity, belonging, and self-esteem (67) of coastal communities, which is clearly seen in MMFR and MWS. Stakeholder efforts in conserving the mangrove forest and promoting ecotourism may further increase the connectivity between MMFR and MWS, and local communities.

The lower score of mental wellbeing in MMFR is attributed to the involvement of respondents in risky fishing activities. Fishing is known to be dangerous, at times, and can be exposed to multiple risks, including weather and sea conditions (12, 68). In addition, intense and prolonged working activity associated with fishing can cause fatigue where such hazardous working conditions become the stress factors in life (69). The association of health-related risks may develop unfavorable outcomes which in turn can increase the impact of psychological stress experienced (69). In contrary to MMFR, MWS has been declared as a conservation area by the City of Surabaya in 2007. Hence, the main industry sector of coastal communities of MWS, ecotourism, is still in its early phase with potential to grow and diversify. The management of MWS has been keeping up with efforts in restoring the mangrove area bringing in more paying visitors to support MWS and local communities (48, 70).

## Challenges and suggestions for improvement

Representatives in the workshops held in both study sites identified several challenges that may jeopardize the benefits of mangrove forests. They also suggested various mitigation measures toward sustainable management of mangrove forests and enhancement of community development. Participants expressed that MMFR was well managed specifically in terms of timber production for charcoal. Despite having good management plans, one of the main concerns regarding the MMFR management plans shared by the participants was risk of infrastructure development and expansion of urbanization in forest areas along with incidences of illegal logging, forest degradation, and funding constraints for forest rehabilitation (Table 4). Representatives also worried about MMFR in favor of development and land use changes. They also highlighted waste management issues, for instance, open dump of garbage into the river and sea, and lack of garbage bin that were available for the local communities and tourists. Participants shared that waste was generated by the villages nearby, and socio-economic activities such as ecotourism, charcoal production, and fisheries were not properly handled by the local communities and authorities. They also reported that community people were not involved with the forestry department to plan/conduct forest management activities. In order to mitigate these challenges, participants suggested several measures (Table 4), which the MMFR authority can consider for further improvement in the management of mangrove forests.

**Table 4:** Challenges of MMFR and MWS identified by workshops participants in Malaysia and Indonesia.

**Challenges**	**Suggestions for improvement**
**MMFR**	
1. Development and urbanization	1. Create inter-agencies collaboration and have a public engagement before the development of policies
2. Illegal logging and forest degradation	2. Planning of land use and proper monitoring by state and local government
3. Risk of forest degazettement	3. Strengthen local community association and their participation in forest management
4. Funding constraints for forest rehabilitation	4. Enforcement toward reducing single-use plastic
5. Lack of direct engagement from local communities in forest management	5. Community engagement in waste management
6. River pollution and lack of awareness in waste management among local communities	6. Create more job opportunities (more products)
7. Erosion of riverbanks	7. Create a National Mangrove Forest Conservation Day
8. Arrival of high volume of tourists causing discomfort to local communities	
9. Migration from Matang to other places to look for alternative income	
**MWS**	
1. Smaller area of MWS and not well known to people	1. Strengthen bonding between government agencies, companies, and local communities
2. Less awareness among local people about mangrove resources and conservation	2. Specialized the agency for appropriate management of MWS resources
3. Lack of funding to maintain/improve the MWS area	3. Provide visitor guide and information
4. Lack of community engagement	4. Install barriers around the mangrove area to protect from rubbish
5. Single agency to manage all resources in MWS	5. Conduct routine monitoring and cleaning the area
6. Educational issue	6. Limitation of food sellers, food place and provide rubbish bins
7. Rubbish and organic waste	7. Create community-based environment awareness and conservation programme

Participants in the Indonesian workshop reported that MWS is a newly declared mangrove forest reserve, and all resources were being managed by a single government agency, the “Food and Agriculture Agency.” Being a new forest reserve, local people were not very much aware about resource conservation, and they were not involved in the management. Participants identified limited funding opportunities for MWS management. They also reported river pollution and lack of educational resources for visitors and local guides as challenges. They suggest various measures for mitigating these issues. Table 4 also shows that there are few common challenges in both mangroves such as lack of funding, community engagement in forest management, and river pollution.

## Practical implications

Mangrove forests have many benefits for coastal communities of the MMFR and MWS in terms of socio-economic opportunities which directly or indirectly impact the subjective and mental wellbeing of coastal communities. Respondents from both study areas identified the development of the tourism sector as one of the primary economic opportunities for local communities with the creation of diverse job opportunities in tourism, accommodation, and catering. Albeit the main income opportunity in MMFR is related to charcoal industries, other opportunities have been identified, including tourism and fisheries. In MWS, the opportunities stemmed from tourism industries where participants worked as staff and food sellers. However, the coastal communities in MMFR and MWS are also aware of challenges that could possess threats to their wellbeing and livelihoods, driven by declining yields for fisheries over the years, rubbish pollution, inconsistencies in the rehabilitation programme for the mangrove forest, and a general lack of mangrove management staff.

## Conclusion

Respondents from both regions have high to average mental wellbeing based on SWEMWBS scores. This outcome shows that the benefits provided by mangrove ecosystems lead to stress reduction when economy resources and job opportunity are secure, and a good mental health of local communities. The difference in the SWEMWBS mean scores between both study sites is rather small among the participants and likely attributed to differences in the nature of industry in the coastal communities (MMFR: charcoal and fisheries industries, MWS: tourism activity and food/restaurant industries). Thus, proper mangrove forest management plays an important role in safeguarding and developing subjective and psychological wellbeing of coastal communities through ensuring the availability of long-term benefits provided by mangrove forests and co-ownership/active engagement in future development plans and implementation.

## Study limitations and future research

One of the main limitations of this study was a small number of interviews, which might affect the generalization of results. Future research may focus on having a larger number of interviews to provide a statistically reliable analysis. Our research only assessed the respondents' perception on benefits and did not quantify the benefits. Adding an economic valuation of ecosystem services would provide a more complete evaluation of mangrove ecosystem services, requiring further studies. In addition, the association between coastal communities' subjective and psychological wellbeing, and the mangrove ecosystem are under study. Such a relationship is pertinent for coastal communities to improve and sustain contentment and life satisfaction. Hence, this study warrants stakeholders and scholars to explore factors that associate for better wellbeing of coastal communities.

## Data availability statement

The original contributions presented in the study are included in the article/supplementary material, further inquiries can be directed to the corresponding authors.

## Ethics statement

The studies involving human participants were reviewed and approved by the Heriot-Watt University, Social Sciences Ethics Committee. The patients/participants provided their written informed consent to participate in this study.

## Author contributions

G-NK, IU, TW, AS, AA, and TN conceived of the idea and apply for research funding. G-NK, IU, TW, AS, AA, TN, JN, and LM developed the workshop and interview materials. G-NK, IU, JN, and LM performed data collection and data analysis. All authors discussed the results and contributed to the final manuscript.

## Funding

This research was funded by Global Challenges Research Fund, The Scottish Funding Council, grant number SFC: P20GCRF7.

## Conflict of interest

The authors declare that the research was conducted in the absence of any commercial or financial relationships that could be construed as a potential conflict of interest.

## Publisher's note

All claims expressed in this article are solely those of the authors and do not necessarily represent those of their affiliated organizations, or those of the publisher, the editors and the reviewers. Any product that may be evaluated in this article, or claim that may be made by its manufacturer, is not guaranteed or endorsed by the publisher.
